# Topological network based drug repurposing for coronavirus 2019

**DOI:** 10.1371/journal.pone.0255270

**Published:** 2021-07-29

**Authors:** Mahnaz Habibi, Golnaz Taheri

**Affiliations:** 1 Department of Mathematics, Qazvin Branch, Islamic Azad University, Qazvin, Iran; 2 Department of Electrical Engineering and Computer Science, KTH Royal Institute of Technology, Stockholm, Sweden; 3 Science for Life Laboratory, Stockholm, Sweden; Alagappa University, INDIA

## Abstract

The COVID-19 pandemic caused by the Severe Acute Respiratory Syndrome Coronavirus 2 (SARS-CoV-2) has become the current health concern and threat to the entire world. Thus, the world needs the fast recognition of appropriate drugs to restrict the spread of this disease. The global effort started to identify the best drug compounds to treat COVID-19, but going through a series of clinical trials and our lack of information about the details of the virus’s performance has slowed down the time to reach this goal. In this work, we try to select the subset of human proteins as candidate sets that can bind to approved drugs. Our method is based on the information on human-virus protein interaction and their effect on the biological processes of the host cells. We also define some informative topological and statistical features for proteins in the protein-protein interaction network. We evaluate our selected sets with two groups of drugs. The first group contains the experimental unapproved treatments for COVID-19, and we show that from 17 drugs in this group, 15 drugs are approved by our selected sets. The second group contains the external clinical trials for COVID-19, and we show that 85% of drugs in this group, target at least one protein of our selected sets. We also study COVID-19 associated protein sets and identify proteins that are essential to disease pathology. For this analysis, we use DAVID tools to show and compare disease-associated genes that are contributed between the COVID-19 comorbidities. Our results for shared genes show significant enrichment for cardiovascular-related, hypertension, diabetes type 2, kidney-related and lung-related diseases. In the last part of this work, we recommend 56 potential effective drugs for further research and investigation for COVID-19 treatment. Materials and implementations are available at: https://github.com/MahnazHabibi/Drug-repurposing.

## Introduction

Recent studies on coronaviruses as a family of positive-strand RNA viruses tried to find that a newly emerged virus belongs to a new or any existing species of this family of viruses. The SARS-CoV-2 as a member of this family differs from SARS-CoV, MERS-CoV, and the other coronaviruses introduced earlier. The available clinical data for SARS-CoV-2 showed that this disease spectrum and transmission differ from SARS-CoV. The wide range of virality and spectrum from asymptomatic to severe and even fatal in some cases for this virus cases that the WHO introduced “Coronovirus disease 19” or “COVID-19” for this virus. [1]. According to the fast spread of SARS-CoV-2, a large number of researchers have been searching for possible treatment for COVID-19 in the past months. At present, no effective medicine has been introduced for the COVID-19 treatment [2]. Timothy et al [3] introduced new oral broad-spectrum antiviral inhibitors for SARS-CoV-2 in humans and multiple coronaviruses in mice.

Producing new drugs with a complete profile is a time-consuming, costly, and challenging procedure. Drug repurposing is one of the effective drug discovery processes from existing drugs. Drug repurposing can help to find new therapies for diseases in a shorter time and with lower costs, especially when the preclinical and safety tests have been completed for drugs [4]. The main purpose of drug repurposing is to increase the therapeutic use of available drugs for more new medical purposes [5]. This process can play a crucial role for patients with rare or complex diseases when there are no other effective treatments are available. Computational methods for drug repurposing offer new testable assumptions for systematic reuse of drugs [6, 7]. It is worth mentioning that, targeting single virus proteins mostly has a high chance of drug resistance as a result of the fast evolution of the virus genome [7].

Viruses need host cellular components for effective replication in the time of infection [7]. Systematic identification of virus-host protein-protein interaction networks provides a productive way to clarify the evolution of virus genomes. Therefore, targeting the virus-host interaction network can offer an innovative strategy for forming effective cures for viral infections like SARS-CoV-2 [8].

Since the outbreak of COVID-19, many research groups have been trying to propose network-based methods to find some effective repurposed drugs to perform against SARS-CoV-2 [9, 10]. Fehr et al [10] revealed that SARS-CoV-2 infects human cells by hijacking the host’s translation mechanism to produce 29 viral proteins. These 29 proteins bind to multiple human proteins to set up the molecular processes needed for viral duplication and additional host infection. Gorden et al [11] proposed a map from human proteins and SARS-CoV-2 proteins that were found to interact in the affinity purification mass spectrum method. They released the 26 proteins from 29 proteins that the SARS-CoV-2 infect in the human body. They also identified 332 human proteins involved in these viral protein binds. From these 332 proteins, they identified 67 druggable human proteins with 69 existing drugs. It is noticeable that the identification of dependency between host proteins and virus infection can provide significant insights to find suitable drug targets for developing antivirals medicine against SARS-CoV-2.

In this paper, we propose a method based on the topological and statistical properties of proteins that are druggable in the human PPI network. We also show the distance between these proteins with the proteins that are hijacked by the virus and measure some properties concerning these measures. In this method, we define 4 informative topological features and 4 informative statistical features for each protein reported by Uniprot as a drug target concerning the status of that protein or their neighbor proteins in our constructed network. Some of the topological features are based on the distance of these proteins from other proteins or hijacked proteins in our constructed PPI network. Some of our defined features are based on the biological properties that are affected by the virus. After defining all of these topological and statistical features, our method clusters the set of 2,898 proteins as drug target proteins in the host cells. The aforementioned method select 800 proteins from these 2,898 proteins with the highest drug-binding capability for COVID-19 treatment. We show that from 17 unapproved drugs used in medical centers for COVID-19 treatment, 15 drugs have at least one target in our selected set. We also show that 281 drugs from 328 drugs are undergoing clinical trials approved with our candidate set. From all of the proteins that are placed in our candidate sets, we find 35 proteins as a final set of disease-associated genes. Our results show that our candidate proteins are targeted by a large number of COVID-19 drugs. In the last part of the results, we also show some significant signaling and disease pathways. Finally, we recommended 56 drugs for more research and investigation that related to the significant disease pathways as candidate drugs for COVID-19 treatment.

## Method

In this section, we define 8 informative topological and statistical features for each protein corresponding to the position of this protein in the PPI network, the position of this protein with respect to the host proteins that are targeted by the virus, and the number of biological processes that this protein participates. Since the problem of finding the appropriate set of drugs for COVID-19 treatment is still an open question, it can be considered as a problem without a response variable or exact answer. Therefore, to find an efficient model, we used only our defined informative topological features for clustering druggable proteins as a suitable candidate set of proteins.

### Databases

#### Protein-Protein Interaction (PPI) network

We use 5 human high-throughput PPI networks in this work. The first one, Huri, contains 52,248 binary interactions [12]. The second one is collected from the Biological General Repository for Interaction Datasets (BioGRID) and contains 296,046 interactions [13]. The BioGRID dataset contains various interactions that are created from different techniques. In this work, we just use the physical interactions between proteins. The three other datasets Human Integrated Protein-Protein Interaction rEference (Hippie) [14], Agile Protein Interactomes Data Server (APID) [15], and Homologous Interactions (Hint) [16] that contain 57,428, 171,448, and 64,399 experimentally validated interactions, respectively. These interactions are derived from high-throughput yeast-two hybrid (Y2H) and mass spectrometry methods. All of the proteins from these five datasets are mapped to their corresponding Universal Protein resource (Uniprot) ID [17]. If a protein could not be mapped to a Uniprot ID, it is removed. The final interactome that we used in this study contains 20,041 proteins and 304,730 interactions. We also use 332 human proteins that interact with 26 proteins of the SARS-CoV-2 virus that reveals in [11].

#### Identification of drugs-human protein interactions

To evaluate our candidate targets, we use all drugs and their corresponding target interactions reported in the Uniprot. These interactions contain 6,163 drugs and 2,898 protein targets. We also use 44 experimental unapproved drugs for COVID-19 treatment reported in DrugBank [18]. From these 44 drugs denoted as Covid-Drug, 27 drugs have no target information and the other 17 drugs have the drug target information. These 17 drugs can target 78 proteins in human cells. The second group of drugs denoted as Clinical-Drug contains 449 drugs as clinical trials for COVID-19 treatment reported in DrugBank [18]. From these 449 drugs, 328 drugs have target proteins in our PPI network. These 328 drugs can target 888 proteins in human cells.

#### Biological process information

We use the information of the biological processes for proteins published on the Gene Ontology (GO) website [19]. We find that 19,439 proteins from these 20,041 proteins or 97% of them are annotated. We use the Informative Biological Process (IBP) concept to avoid achieving the incorrect conclusions caused by biases in the annotation process. We consider the IBP annotations if it has two properties. First, it needs to have at least *k* proteins annotated with it. Second, each of its descendant’s GO terms needs to have less than *k* proteins annotated with them. In this study, we set 3 as a value of *k*. We note that 16,021 biological processes corresponding to these 20,041 proteins are participating in our interactions. From these 16,021 biological processes, 1,374 IBP GO terms affected by the virus in which a subset of 332 host proteins as possible targets of the virus is involved [11].

We also define the overlap between two biological process *p*_1_ and *p*_2_ in the following way (|.| denotes the size):

$$\begin{array}{c} Overlap\left(p_{1},p_{2}\right)=\frac{|p_{1}⋂p_{2}{|}^{2}}{|p_{1}\left|\right|p_{2}|}. \end{array}$$ (1)

Finally, we removed the processes with more than 15% overlaps. With this filtering method, we have 1,213 non-overlapping biological processes corresponding to SARS-CoV-2.

### Representative criteria

Each PPI network is considered as an undirected graph, *G* = (*V*, *E*), where *V* = {*v*_1_, *v*_2_, …, *v*_*n*_} is a set of vertices represent proteins in *G* and *e*_*ij*_ ∈ *E* is the set of edges represents a functional interaction between *v*_*i*_ and *v*_*j*_. We call two vertices *v*_*i*_ and *v*_*j*_ as neighbors if there is an edge between them. Suppose *N*(*v*_*i*_) is a set of all neighbors for a vertex *v*_*i*_, therefore *d*(*v*_*i*_) = |*N*(*v*_*i*_)| shows the degree of *v*_*i*_. A path between two vertices *v*_*i*_ and *v*_*j*_ is a sequence of edges that connects a sequence of distinct vertices (*v*_*i*_ = *v*_0_, *v*_1_, …, *v*_*n*_ = *v*_*j*_) and the number of edges in each path is defined as path length. The shortest path between two vertices *v*_*i*_ and *v*_*j*_ is defined as a path with the minimum length that is indicated by *d*(*v*_*i*_, *v*_*j*_).

We define two groups of characteristics associated with a graph *G* = (*V*, *E*). The first group of characteristics $\rho_{G}:V\to R$, depends only on the graph topological properties. The group *ρ*_*G*_, contains 4 informative topological features for each protein reported in Uniprot as a drug target and their interaction with the virus. The second group of characteristics $\rho_{G,S}:V\to R$, depends on a set *S* that represents statistical information about COVID-related drugs and biological processes affected by the virus.

#### Topological features

The following four properties show the informative topological features in the group *ρ*_*G*_, for each protein reported in Uniprot as a drug target.

1. *DR*(*v*_*i*_): The ratio of the number of neighbors for each protein *v*_*i*_ in the PPI network that is targeted by virus proteins.
  $$\begin{array}{c} DR\left(v_{i}\right)=\frac{\left|N(v_{i})⋂T\right|}{d(v_{i})}. \end{array}$$ (2)
  The set, *T*, shows 332 proteins as possible targets of the virus and |.| indicates the size of a set. The larger value of *DR*(*v*_*i*_), indicates that the high ratio of virus’s neighbors are being targeted.
2. *AN*(*v*_*i*_): The average ratio of the number of neighbors for each protein *v*_*i*_ [20].
  $$\begin{array}{c} AN\left(v_{i}\right)=\frac{\sum_{v_{j}\in N\left(v_{i}\right)}d\left(v_{j}\right)}{d(v_{i})}. \end{array}$$ (3)
  A large value for the average degree of neighbors of each protein, *v*_*i*_, indicates the presence of essential proteins in its neighborhood.
3. *D*(*v*_*i*_, *T*): The minimum distance of each protein *v*_*i*_ from all of the vertices of set *T*. The smaller value of this distance indicates the closeness of vertex *v*_*i*_ to set *T*.
4. *MD*(*v*_*i*_): The average of minimum distance between each protein *v*_*j*_ in the vertex’s neighborhood and set *T*.
  $$\begin{array}{c} MD\left(v_{i}\right)=\frac{\sum_{v_{j}\in N\left(v_{i}\right)}D\left(v_{j},T\right)}{d(v_{i})}. \end{array}$$ (4)
  The smaller value of mean distance of the neighbors of each protein indicates the closeness of the neighbors of this vertex to *T*.

#### Statistical features

Let *S* represents information about COVID-related drugs and biological process for each protein reported in Uniprot as a drug target. The following properties show the informative statistical features in the group *ρ*_*G*,*S*_, for each protein.

1. Suppose that *π* = {*p*_1_, *p*_2_, …, *p*_*k*_} shows the non-overlapping biological processes corresponding to the virus. We define the number of non-overlapping biological processes which the protein, *v*_*i*_, is involved as follow:
  $$\begin{array}{c} IBP\left(v_{i}\right)=\left|\left\{p_{j}\in \pi |v_{i}\in p_{j}\right\}\right|. \end{array}$$ (5)
  The larger value of *IBP*(*v*_*i*_) indicates that protein *v*_*i*_ is valuable in terms of participating in a further number of biological processes.
2. Suppose that *π* = {*p*_1_, *p*_2_, …, *p*_*k*_} shows the non-overlapping biological processes corresponding to the virus. We define the participation rate of each protein, *v*_*i*_, in set *π* as follow:
  $$\begin{array}{c} P_{IBP}\left(v_{i}\right)=1-\sum_{p_{i}\in \pi }(\frac{|N\left(v_{i}\right)⋂p_{i}|}{\sum_{p_{i}\in \pi }|N\left(v_{i}\right)⋂p_{i}|}{)}^{2}. \end{array}$$ (6)
  The possible values for *P*_*IBP*_(*v*_*i*_) is between 0 and 1. The closer value of *P*_*IBP*_(*v*_*i*_) to 1 shows the distribution of the neighbors of this vertex in the set of biological processes [21].
3. *COV*(*v*_*i*_): Indicates the number of drugs in a Covid-Drug group and have targeted protein *v*_*i*_.
4. *CID*(*v*_*i*_): Indicates the number of drugs in Clinical-Drug group and have targeted protein *v*_*i*_.

### Suggesting the appropriate drugs for COVID-19 treatment

In this subsection, we design a two-step method to find an effective solution for the COVID-19 treatment problem. In the first step to point out some appropriate COVID-19 associated genes, we define 8 topological and statistical features. In the second step, we narrow down these associated genes by considering some of them that contributed to the COVID-19 comorbidities. These comorbidities can affect the severity of COVID-19. Finally, we suggest a set of FDA-approved drugs related to disease pathways with respect to these disease-associated genes as candidate drugs for more investigation as COVID-19 treatment.

#### Finding candidate set of target proteins related to COVID-19

Suppose that a set *δ* includes all the drugs in the Uniprot that target human proteins. Also assume that a set *τ* = {*v*_1_, …, *v*_*m*_} includes a set of human proteins that is targeted by a drug set *δ*. Now for each specific topological feature, *ρ* = *ρ*_*G*_ and for each specific statistical feature, *ρ* = *ρ*_*G*,*S*_, we define a numerical set *ρ*(*τ*) = {*ρ*(*v*_1_), …, *ρ*(*v*_*m*_)} with mean value $\bar{\rho }$.

Suppose that *φ*_1_ contains a set of statistical and topological features (*DR*(*v*_*i*_) and *AN*(*v*_*i*_) as topological features), then for each protein *v*_*i*_ ∈ *τ* we define the following measure.

- *a*(*v*_*i*_): Number of features such that *ρ* ∈ *φ*_1_ and $\rho \left(v_{i}\right)>\bar{\rho }$.

Suppose that *φ*_2_ is a set contains two topological features *D*(*v*_*i*_, *T*) and *MD*(*v*_*i*_), then for each *v*_*i*_ ∈ *τ* we define the following measure.

- *b*(*v*_*i*_): Number of features such that *ρ* ∈ *φ*_2_ and $\rho \left(v_{i}\right)<\bar{\rho }$.

Now for each *v*_*i*_ ∈ *τ* we define a new measure *s*(*v*_*i*_) = *a*(*v*_*i*_) + *b*(*v*_*i*_), then three candidate sets *T*_1_, *T*_2_ and *T*_3_ define as follow.

$$\begin{array}{c} T_{1}=\left\{v\in \tau |s\left(v\right)>4\right\}.\\ T_{2}=\left\{v\in \tau |s\left(v\right)>5\right\}.\\ T_{3}=\left\{v\in \tau |s\left(v\right)>6\right\}. \end{array}$$

It is noticeable that the large value corresponding to the features in *φ*_1_ and the small value corresponding to the features in *φ*_2_ indicate that a vertex is valuable. As a result, set *T*_1_ contains proteins that have at least five valuable feature, set *T*_2_ contains proteins that have at least six valuable features and set *T*_3_ contains proteins that have at least seven valuable features from eight features.

#### Finding disease-associated genes and related drugs

The results of the previous subsection can be nominated as suitable candidate sets of proteins with important biological roles. It is noticeable that not all of the selected proteins are appropriate candidates as a drug target for the COVID-19. Therefore, we narrow down these candidate proteins to the disease-associated genes. Different patients with COVID-19 show various symptoms from asymptomatic to death. The severity and death in patients with COVID-19 are related to neutrophils proliferation elevation and reduction in lymphocytes population (lymphopenia) in patients [22]. It is noticeable that patients with underlying diseases such as cardiovascular diseases, diabetes, hepatitis, lung diseases, kidney disease, and different cancer types have more severe symptoms than others. Therefore, we correlate the genes associated with these mentioned diseases with the genes associated with COVID-19 pathology. To identify a set of disease-associated genes related to COVID-19 as drug targets, we study the subset of genes that are associated with the aforementioned diseases in our candidate set. We use gene-disease relation from Database for Annotation, Visualization, and Integrated Discovery (DAVID) to find these disease-associated genes. We select proteins that are corresponding to four out of five of these specific comorbid diseases with a significant p-value. We also specify the significant disease-pathway enrichments for our selected disease-associated genes from DAVID tools. Then, we characterize significant disease-pathways with a p-value less than 0.06 and detect FDA-approved treatments for the significant disease-pathways from FDA and Mayo Clinic databases (https://www.mayoclinic.org).

## Results

In this section, we evaluate the candidate drug targets from different perspectives and suggest some appropriate candidate drugs for COVID-19 treatment.

### Evaluation of candidate sets

#### Statistical properties of candidate sets

In the previous section, we introduced three sets *T*_1_, *T*_2_, and *T*_3_ as candidate drug targets for COVID-19. Table 1 presents some statistical properties of these sets and set *τ* respectively. The first column shows the number of vertices for each selected set and the total number of vertices for set *τ*. The next columns show the average of the values obtained for each feature. Table 1 shows that, on average, each vertex in the sets *T*_1_, *T*_2_, and *T*_3_ participates in 5.06, 6.31, and 7.48 biological processes affected by the virus, respectively. On average, each vertex in set *τ* is located at a distance of 1.54 from the target of the virus (*T*). While each vertex in the selected set *T*_3_ is located at a distance of 0.921 from the set *T*.

**Table 1:** The first column shows the number of proteins for each selected set and the total number of vertices for set *τ*. The next columns show the average values obtained for each feature in each of the selected set.($\bar{X}$ denotes the average values).

	No. Proteins	$\bar{P_{IBP}\left(v_{i}\right)}$	$\bar{MD(v_{i})}$	$\bar{D(v_{i},T)}$	$\bar{AN(v_{i})}$	$\bar{DR(v_{i})}$	$\bar{IBP(v_{i})}$	$\bar{CoD(v_{i})}$	$\bar{ClD(v_{i})}$
*T*_1_	800	0.986	1.11	0.967	169.1	0.06	5.06	0.1175	1.753
*T*_2_	260	0.987	1.09	0.962	196.59	0.066	6.31	0.303	4.07
*T*_3_	64	0.989	1.07	0.921	210.92	0.068	7.48	0.328	4.42
*τ*	2898	0.917	1.03	1.54	167.3	0.027	3.1	0.05	0.997

**Fig 1:**
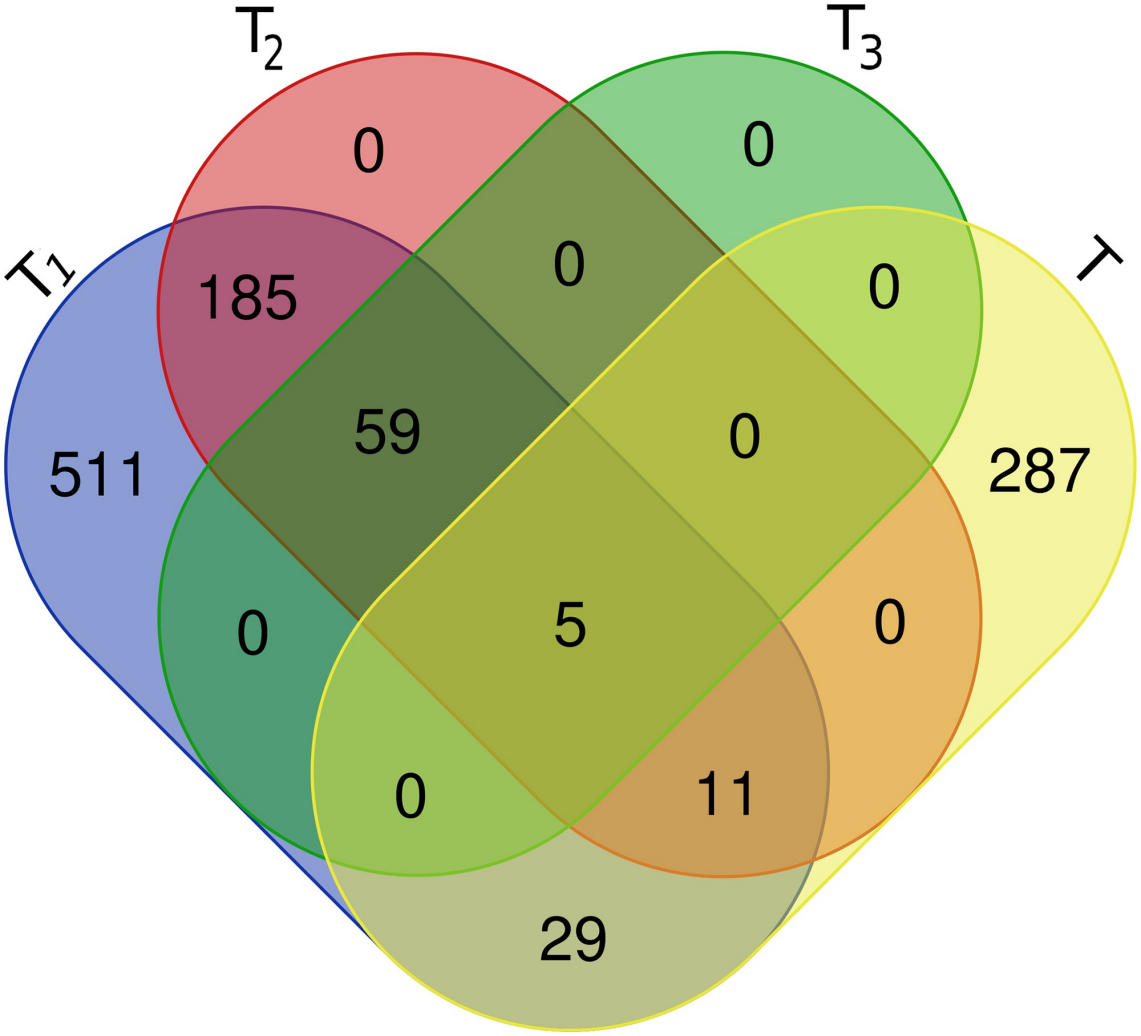
The Venn diagram shows the relation of vertices of the candidate sets and set *T*.

The Venn diagram in Fig 1 illustrates the relation of vertices for the candidate sets and set *T*. Fig 1 shows that 45 proteins from set *T*_1_, 16 proteins from the set *T*_2_, and 5 protein from the set *T*_3_ interact with the virus proteins. In general, the above statistical results show that our selected sets include a part of proteins that directly interact with virus proteins. These selected sets also include proteins that are topologically and statistically important and valuable.

### Evaluation of candidate sets with respect to random sets

In order to evaluate candidate sets, we compare sets *T*_1_, *T*_2_ and *T*_3_ with randomly generated subsets of set *τ*. For each set with known number of vertices *n*, we have selected 10^3^ randomly generated sets from set *τ* as a sample drug target set. Suppose that *N*_*i*_ and *M*_*i*_ for *i* = 1, …, 10^3^ denotes the number of Covid-Drug and Clinical-Drug in *i*-th randomly generated sets in group of size *n*, respectively. Assume that *N*_*co*_ and *N*_*cl*_ show the number of drugs in Covid-Drug and Clinical-Drug groups, respectively, that are approved by our selected set. Now suppose that *X*_*co*_ = {*i*|*N*_*i*_ > *N*_*co*_} and *X*_*cl*_ = {*i*|*M*_*i*_ > *N*_*cl*_} for *i* = 1, …, 10^3^ denote the random sets that performed better than the proposed sets. The null hypothesis, *H*_0_, is that our selected drug set of size *n* is not important. The alternative hypothesis, *H*_1_, is that our selected drug set of size *n* is indeed important. We use Exceeding Value (EV) for Covid-Drug and Clinical-Drug as:

$$\begin{array}{c} EV_{co}=\frac{|X_{co}|}{1000},\\ EV_{cl}=\frac{|X_{cl}|}{1000}, \end{array}$$

where |*X*| denotes the size of *X*. If *EV*_*co*_(*EV*_*cl*_) < *α* then, we reject *H*_0_ (*α* is a threshold value that we consider to be 0.05). The values of *EV*_*co*_(*EV*_*cl*_) for three selected drug sets are reported in Table 2 (These values cause extremely significant results). We can conclude that our selected sets show a better performance than all of these random sets.

**Table 2:** The values of *EX*_*co*_ and *EX*_*cl*_ for three selected drug sets.

	*T*_1_	*T*_2_	*T*_2_
*X*_*co*_	5	11	45
*X*_*cl*_	0	0	28
*EV*_*co*_	0.005	0.011	0.045
*EV*_*cl*_	0	0	0.028

In Table 3 for each of the proposed sets, we compare the mean value of each feature for the two groups of random sets *X*_*co*_ and *X*_*co*′_ = {*i*|*N*_*i*_ <= *N*_*co*_} as well as the random sets *X*_*cl*_ and *X*_*cl*′_ = {*i*|*M*_*i*_ <= *N*_*cl*_}. Table 3 shows the average value for each feature for two mentioned groups of random sets. The first group contains random sets that perform better than our candidate sets in terms of the number of Covid-Drugs and Clinical-Drugs. The second group contains random sets that do not perform better than our candidate sets in terms of the number of Covid-Drugs and Clinical-Drugs. Comparison of these two groups of sets shows that the random sets, which include more Covid-Drugs and Clinical-Drugs (*X*_*co*_ and *X*_*cl*_), contain proteins that are more valuable and important in terms of topological and statistical properties. It is noticeable that the comparison of Tables 3 and 1 does not indicate the superiority of the random sets *X*_*co*_ and *X*_*cl*_ compared to our candidate sets in terms of having more valuable proteins with respect to the topological and statistical features.

**Table 3:** The first column shows two groups of random sets in each selected set. The next columns show the average values obtained for each feature in each of the selected set. ($\bar{X}$ denotes the average values).

*T*_1_	$\bar{P_{IBP}\left(v_{i}\right)}$	$\bar{MD(v_{i})}$	$\bar{D(v_{i},T)}$	$\bar{AN(v_{i})}$	$\bar{DR(v_{i})}$	$\bar{IBP(v_{i})}$	$\bar{CoD(v_{i})}$	$\bar{ClD(v_{i})}$
*X*_*co*_	**0.918**	**1.296**	**1.542**	166.554	**0.028**	**3.217**	**0.062**	**1.193**
*X*_*co*′_	0.9067	1.303	1.547	**167.241**	0.026	3.015	0.050	1.003
*T*_2_	$\bar{P_{IBP}\left(v_{i}\right)}$	$\bar{MD(v_{i})}$	$\bar{D(v_{i},T)}$	$\bar{AN(v_{i})}$	$\bar{DR(v_{i})}$	$\bar{IBP(v_{i})}$	$\bar{CoD(v_{i})}$	$\bar{ClD(v_{i})}$
*X*_*co*_	**0.991**	**1.403**	1.557	**167.588**	**0.029**	3.094	**0.101**	**1.555**
*X*_*cl*_	0.817	1.302	**1.541**	160.301	0.017	**3.102**	0.049	0.982
*T*_3_	$\bar{P_{IBP}\left(v_{i}\right)}$	$\bar{MD(v_{i})}$	$\bar{D(v_{i},T)}$	$\bar{AN(v_{i})}$	$\bar{DR(v_{i})}$	$\bar{IBP(v_{i})}$	$\bar{CoD(v_{i})}$	$\bar{ClD(v_{i})}$
*X*_*co*_	**0.931**	**1.103**	**1.554**	**167.630**	**0.033**	**3.245**	**0.221**	**2.855**
*X*_*co*′_	0.916	1.304	1.475	165.534	0.027	3.118	0.044	0.951
*X*_*cl*_	**0.938**	1.303	**1.431**	**167.561**	**0.038**	**3.235**	**0.022**	**3.039**
*X*_*cl*′_	0.916	**1.304**	1.543	165.807	0.027	3.111	0.047	0.973

**Fig 2:**
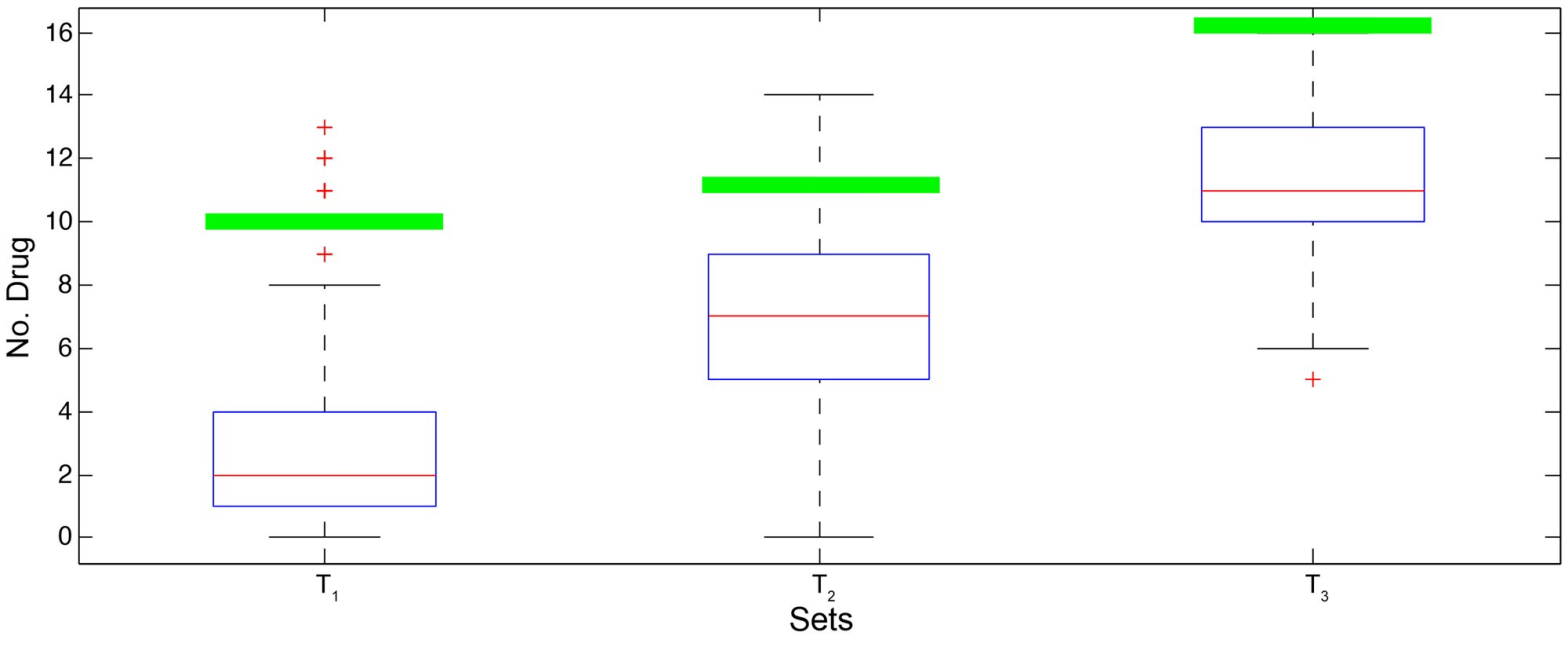
The boxplot of the results of the random sets for Covid-Drug.

**Fig 3:**
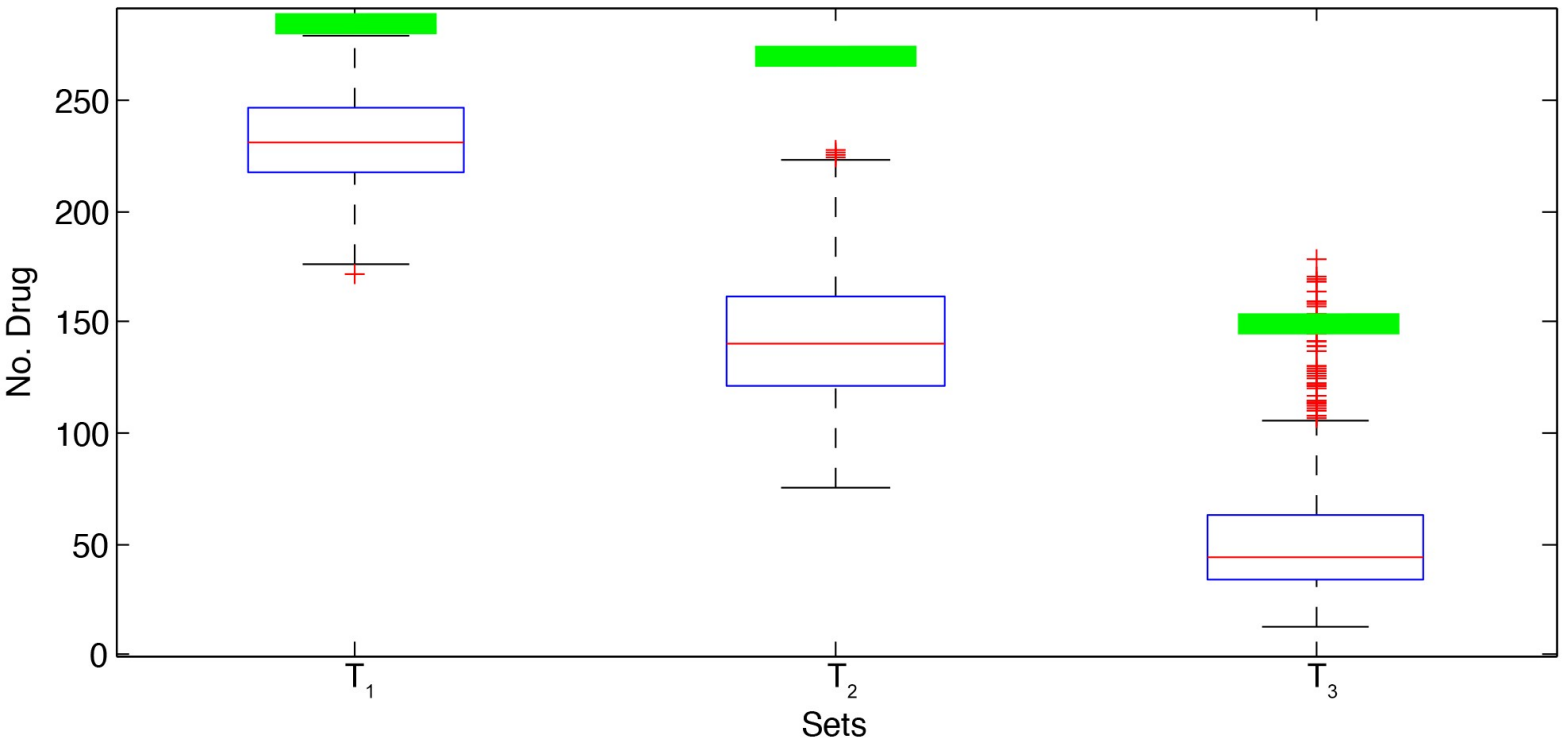
The boxplot of the results of the random sets for Clinical-Drug.

In addition, Figs 2 and 3 demonstrate the boxplot of the results of the random sets for each drug set. The green line in each boxplot shows the number of drugs in Covid-Drug and Clinical-Drug that are approved by our selected set respectively. These two figures show that the results of our selected sets are significantly better than random sets. It means that random sets can not have acceptable results in comparison to our selected sets, and our results are completely different from the random sets.

### Evaluation of candidate sets with respect to the number of approved drugs

Since there is no specific and exact set of drugs for COVID-19, we used two groups Covid-Drug and Clinical-Drug to evaluate the obtained candidate sets. The first group includes unconfirmed drugs used in medical centers for COVID-19 (Covid-Drug) and the second group includes recommended drugs that are currently in clinical trials (Clinical-Drug). In addition, we used another group of drugs to find some appropriate drugs for COVID-19 base on candidate target sets. This group of drugs (All-drug) includes all the drugs available on the Uniprot site. Table 4 provides a comparison between the proteins of three candidate sets as target proteins and the proteins that are targeted by these three groups of drugs (Covid-Drug, Clinical-Drug, and All-drug). The first row presents the total number of proteins targeted by these three groups of drugs. The number of proteins targeted by these drugs in sets *T*_1_, *T*_2_, and *T*_3_ are reported in the second, third, and fourth rows respectively. The ratio of the number of targets presented in the second, third, and fourth rows to the total number of proteins targeted by these three groups of drugs reported in the fifth, sixth, and seventh rows respectively. As shown in Table 5, we also evaluate the number of approved drugs in three groups of drugs. In Table 5, the first row presents the total number of drugs in each candidate for three groups of drugs. The number of drugs approved by these drugs in sets *T*_1_, *T*_2_, and *T*_3_ are reported in the second, third, and fourth rows respectively. The ratio of the number of approved drugs presented in the second, third, and fourth rows to the total number of drugs in these three groups of drugs reported in the fifth, sixth, and seventh rows respectively.

**Table 4:** The number of protein targets in each candidate set for All-drug, Clinical-Drug, and Covid-Drug groups reported in the four first rows. The ratio of the number of targets presented in the second, third, and fourth rows to the total number of proteins targeted by these three groups of drugs reported in the fifth, sixth, and seventh rows respectively.

	Covid-Drug	Clinical-Drug	All-Drug
*τ*	78	888	2898
*T*_1_	42	337	800
*T*_2_	31	187	260
*T*_3_	14	64	64
Ratio of *T*_1_ to all	**0.5385**	**0.379**	**0.276**
Ratio of *T*_2_ to all	0.397	0.2103	0.0897
Ratio of *T*_3_ to all	0.179	0.072	0.221

**Table 5:** The number of drugs in each candidate set for All-drug, Clinical-Drug, and Covid-Drug groups reported in the four first rows. The rate of the number of drugs presented in the second, third, and fourth rows to the total number of drugs in each group reported in the fifth, sixth, and seventh rows respectively.

	Covid-Drug	Clinical-Drug	All-Drug
*δ*	17	328	6163
*T*_1_	15	281	3721
*T*_2_	13	251	2500
*T*_3_	9	138	138
Ratio of *T*_1_ to all	**0.833**	**0.856**	**0.603**
Ratio of *T*_2_ to all	0.722	0.765	0.4056
Ratio of *T*_3_ to all	0.5	0.42	0.022

### Evaluation of candidate genes associated with COVID-19 pathology

Evaluations of the previous subsection showed that set *T*_1_ with 800 proteins has the highest drug approval rate among the two Covid-Drug and Clinical-Drug groups. To identify disease-associated genes as drug targets related to COVID-19, we study the subset of disease-associated genes correlated with mentioned diseases in the selected set (*T*_1_). Set *E* contains the 35 proteins annotated to four out of five of these specific comorbid diseases in *T*_1_. These proteins are selected with respect to a significant p-value obtained by DAVID tools. Table 6 shows these disease-associated genes that are related to COVID-19 pathology. We find that from 17 drugs in Covid-Drug, 11 drugs including **Azithromycin, Bevacizumab, Chloroquine, Colchicine, Darunavir, Dexamethasone, Fingolimod, Ibuprofen, Methylprednisolone, Ritonavir**, and **Tocilizumab** are approved by set *E*. We also find that from 328 drugs in the Clinical-Drug group 179 drugs are approved by set *E*.

**Table 6:** Disease genes in set *E* associated with COVID-19 pathology.

Uniprot ID	Gene name	Uniprot ID	Gene name	Uniprot ID	Gene name
O14746	TERT	P00533	EGFR	P00734	F2
P01130	LDLR	P01375	TNF	P02649	APOE
P02751	FN1	P03372	ESR1	P11021	HSPA5
P04179	SOD2	P04637	TP53	P05181	CYP2E1
P05362	ICAM1	P08684	CYP3A4	P09211	GSTP1
P09601	HMOX1	P10415	BCL2	P10635	CYP2D6
P15692	VEGFA	P16410	CTLA4	P16860	NPPB
P28482	MAPK1	P29460	L12B	P29474	NOS3
P31749	AKT1	P35228	NOS2	P35354	PTGS2
P35568	IRS1	P38936	CDKN1A	P40763	STAT3
P42345	MTOR	P48357	LEPR	P78527	PRKDC
Q8WTV0	SCARB1	Q9NR96	TLR9		

We study the signaling-pathway enrichments identified by bio-pathway DAVID tools related to 35 disease-associated genes (*E* set). Table 7 shows the top significantly enrichment signaling pathways. These pathways have a significant p-value (less than 0.06). Some of these pathways enrichment related to COVID-19 like *(HIF-1, PI3K-Akt)* have been introduced in the other studies [23, 24].

**Table 7:** Some of the significantly enrichment signaling pathways associated with COVID-19.

**Annotation Cluster 1 (Enrichment Score: 3.32)**		
**Term**	**Associated proteins**	**P-value**
hsa04066:HIF-1 signaling pathway	P42345, P29474, P15692, P10415, P28482, P31749, P38936, P00533, P09601, P35228, P40763	1.73E-11
hsa04151:PI3K-Akt signaling pathway	P42345, P29474, P15692, P10415, P04637, P28482, P31749, P38936, P00533, P35568, P02751	3.94E-06
hsa04068:FoxO signaling pathway	P28482, P31749, P38936, P00533, P35568, P40763, P04179	4.19E-05
hsa04150:mTOR signaling pathway	P42345, P28482, P31749, P35568, P01375	1.75E-04
hsa04370:VEGF signaling pathway	P29474, P15692, P28482, P31749, P35354	2.13E-04
hsa04071:Sphingolipid signaling pathway	P29474, P10415, P04637, P28482, P31749, P01375	2.76E-04
hsa04012:ErbB signaling pathway	P42345, P28482, P31749, P38936, P00533	8.29E-04
hsa04915:Estrogen signaling pathway	P29474, P28482, P31749, P03372, P00533	0.001344
hsa04919:Thyroid hormone signaling pathway	P42345, P04637, P28482, P31749, P03372	0.002334
hsa04722:Neurotrophin signaling pathway	P10415, P04637, P28482, P31749, P35568	0.002726
hsa04921:Oxytocin signaling pathway	P29474, P28482, P38936, P00533, P35354	0.006057
hsa04022:cGMP-PKG signaling pathway	P16860, P29474, P28482, P31749, P35568	0.007266
hsa04910:Insulin signaling pathway	P42345, P28482, P31749, P35568	0.030046
hsa04010:MAPK signaling pathway	P04637, P28482, P31749, P00533, P01375	0.034867
**Annotation Cluster 2 (Enrichment Score: 2.71)**		
**Term**	**Associated proteins**	**P-value**
hsa04920:Adipocytokine signaling pathway	P42345, P31749, P48357, P35568, P01375, P40763	2.09E-05
**Annotation Cluster 3 (Enrichment Score: 2.59)**		
**Term**	**Associated proteins**	**P-value**
hsa04620:Toll-like receptor signaling pathway	P28482, P31749, P29460, Q9NR96, P01375	0.001731
hsa04668:TNF signaling pathway	P28482, P31749, P05362, P35354, P01375	0.001792
hsa04660:T cell receptor signaling pathway	P16410, P28482, P31749, P01375	0.012871
**Annotation Cluster 4 (Enrichment Score: 2.23)**		
**Term**	**Associated proteins**	**P-value**
hsa04917:Prolactin signaling pathway	P28482, P31749, P03372, P40763	0.005014

**Fig 4:**
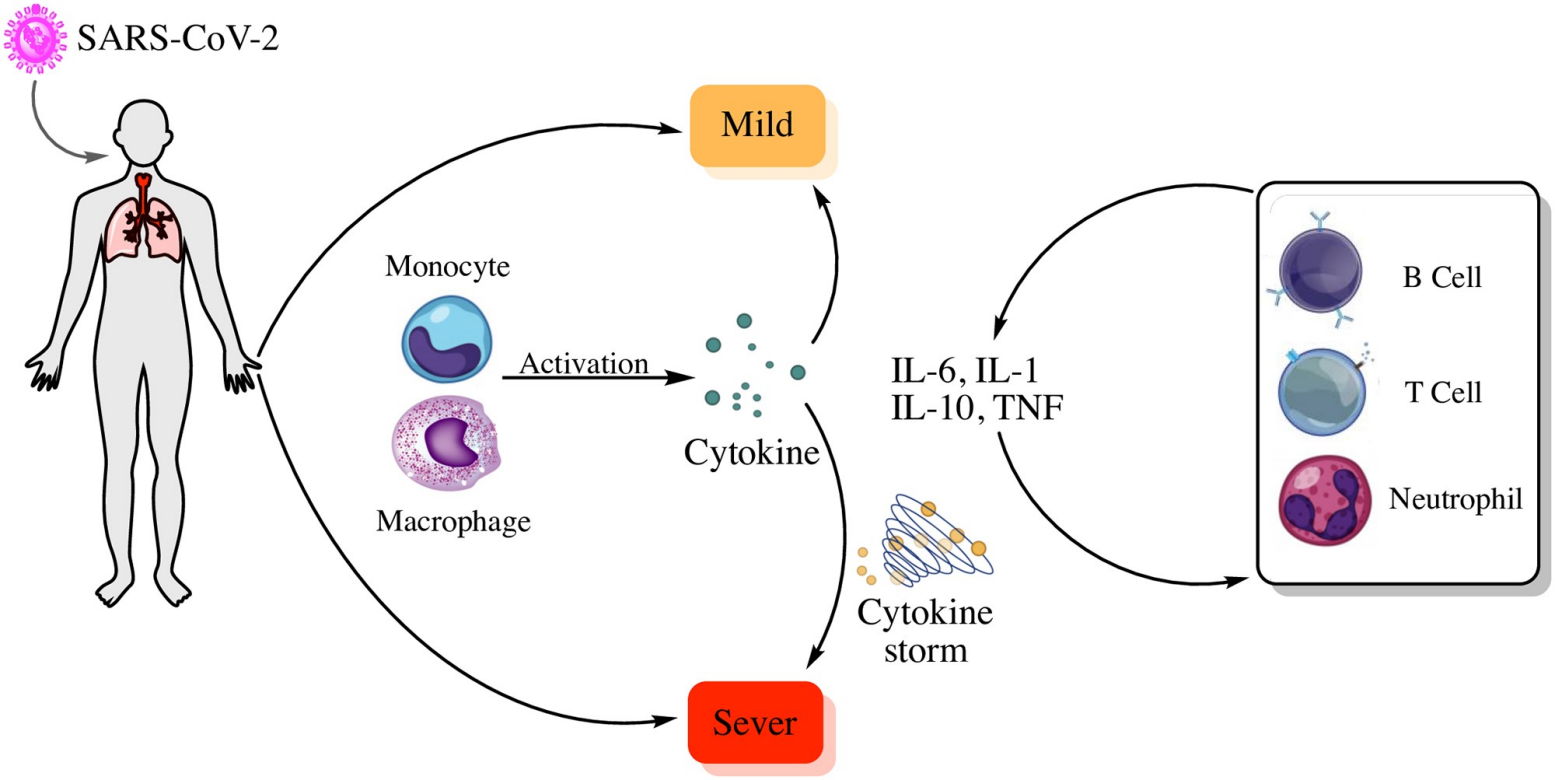
Schematic illustration of SARS-CoV-2 infection and the role of *HIF* − 1*α* on SARS-CoV-2 pathogenesis.

One of the most significant signaling pathways in our results is the *HIF* − 1 signaling pathway. This pathway plays an important role as the first reaction of the body upon pathogens. This pathway and its downstream signaling cascade have a major role in the dominant response of innate immunity against infection. The innate immune response to pathogens is related to some important immune cells like neutrophils and macrophages. The hyperactivity of these cells can drive the production of a high amount of inflammatory cytokines or “cytokine storm” in the region of infection. It is noticeable that the previous studies showed that SARS-CoV-2 result in a high inflammatory response and cytokine storm in severe cases. *HIF* − 1*α* has a major role in response to the hypoxia microenvironment in the site of inflammation. It works as a main regulator in the phagocytes. It can increase the inflammatory response with up-regulation of the angiogenesis factors like VEGF. Therefore, *HIF* − 1*α* inhabitation with pharmacological strategies might introduce a new approach for COVID-19 treatment. It is worth mentioning that *HIF* − 1*α* has a positive impact on the autophagy process. It can suppress the viral infection of SARS-CoV-2 in the host cells and decrease the virus proliferation [23, 24]. Fig 4 shows the schematic illustration of SARS-CoV-2 infection and the role of *HIF* − 1*α* on SARS-CoV-2 pathogenesis. This Figure shows that after infection, the *HIF* − 1*α* induction in the severe situation. In this situation, the inflammatory condition stabilizes inflammatory cells like macrophages and neutrophils. It also enforces cytokine production by these cells and makes cytokine storms.

We analyze the significant disease-pathway enrichments for the candidate proteins related to COVID-19 (set *E*). We also investigate FDA-approved treatments for the significant disease-pathways. In Table 8, we report some of these significant disease-pathways like *(Hepatitis C, Influenza A, Tuberculosis)* that have significant p-values. These pathways contain disease-associated genes that are reported through our method. Some of these drugs like *Cyclosporine, Enzalutamide*, and *Imatinib* are undergoing clinical trials. From 56 drugs reported in Table 8, 26 drugs are reported in other studies as possible candidates for COVID-19 drug repurposing. The other drugs that are reported through our method, can be suitable candidates for more investigation in clinical trials for COVID-19 treatment.

**Table 8:** Some of the significant disease-pathways associated with COVID-19. (Drugs in Clinical-Drug group are highlighted in bold font).

**Annotation Cluster 1 (Enrichment Score: 3.32)**				
**Term**	**Associated proteins**	**P-value**	**FDA Approved**	**Reference**
hsa05205:Proteoglycans in cancer	P42345, P15692, P04637, P28482, P29460, P38936, P03372, P00533, P01375, P40763, P31749, P02751	1.52E-09	Vismodegib	[25]
			Afuresertib	
			Afuresertib hydrochloride	
			Dacomitinib	
			Necuparanib	
			Lumretuzumab	
hsa05200:Pathways in cancer	P10415, P04637, P28482, P31749, P35354, P35228, P42345, P15692, P09211, P02751, P40763, P38936, P00533	1.78E-07	glucocorticoid	
			**Cyclosporine**	[18, 26]
hsa05160:Hepatitis C	P01130, P04637, P28482, P31749, P38936, P00533, P01375, P40763 Q8WTV0	1.88E-07	Interferon	
			**Ribavirin**	[18, 27]
hsa05215:Prostate cancer	P42345, P10415, P04637, P28482, P31749, P38936, P00533	3.72E-06	**Enzalutamide**	[18, 26, 28]
hsa05206:MicroRNAs in cancer	P42345, P15692, P10415, P04637, P00533, P35354, P09601, P35568 P38936, P40763	6.86E-06	**Decitabine**	[18]
hsa05212:Pancreatic cancer	P15692, P04637, P28482, P31749, P40763, P00533	1.45E-05	Erlotinib hydrochloride	
hsa05214:Glioma	P42345, P04637, P28482, P31749, P38936, P00533	1.45E-05	Disopyramide	
hsa05219:Bladder cancer	P15692, P04637, P28482, P38936, P00533	4.43E-05	Erdafitinib	
hsa05222:Small cell lung cancer	P10415, P04637, P31749, P35354, P35228, P02751	5.37E-05	Trilaciclib	
hsa05161:Hepatitis B	P10415, P04637, P28482, P31749, P38936, P01375, P40763	6.54E-05	Peginterferon alfa-2a	
hsa05230:Central carbon metabolism in cancer	P42345, P04637, P28482, P31749, P00533	2.56E-04	Pralsetinib	
			Selpercatinib	
			Telaglenastat	
hsa05218:Melanoma	P04637, P28482, P31749, P38936, P00533	3.82E-04	Trametinib	[29]
			Encorafenib	[30]
hsa05221:Acute myeloid leukemia	P42345, P28482, P31749, P40763	0.0025575	Midostaurin	[31]
			Gilteritinib fumarate	
hsa05223:Non-small cell lung cancer	P04637, P28482, P31749, P00533	0.0025575	Gefitinib	[28]
			Erlotinib hydrochloride	[28]
			Crizotinib	[28]
hsa05210:Colorectal cancer	P10415, P04637, P28482, P31749	0.0034196	5-Fluorouracil	
			Capecitabine	
hsa04210:Apoptosis	P10415, P04637, P31749, P01375	0.0034196	Etanercept	[32]
			Lenalidomide	[31]
			Talazoparib	
hsa05220:Chronic myeloid leukemia	P04637, P28482, P31749, P38936	0.0052143	**Imatinib**	[18]
hsa05014:Amyotrophic lateral sclerosis (ALS)	P10415, P04637, P01375	0.0250501	Edaravone	[34]
			Riluzole	
**Annotation Cluster 2 (Enrichment Score: 2.71)**				
**Term**	**Associated proteins**	**P-value**	**FDA Approved**	**Reference**
hsa04930:Type II diabetes mellitus	P42345, P28482, P35568, P01375	0.0016394	**Metformin**	[18]
			Sulfonylureas	[35]
			DPP4 inhibitors	[36]
**Annotation Cluster 3 (Enrichment Score: 2.59)**				
**Term**	**Associated proteins**	**P-value**	**FDA Approved**	**Reference**
hsa05145:Toxoplasmosis	P01130, P10415, P28482, P31749, P29460, P35228, P01375, P40763	8.32E-07	Pyrimethamine	
			Sulfadiazine	
hsa05152:Tuberculosis	P10415, P28482, P31749, P29460, Q9NR96, P35228, P01375	1.97E-04	Isoniazid	[33]
			Rifampin	[33]
hsa05140:Leishmaniasis	P28482, P29460, P35354, P35228, P01375	3.82E-04	Amphotericin B	
			miltefosine	
hsa05143:African trypanosomiasis	P29460, Q9NR96, P05362, P01375	5.44E-04	Pentamidine	[33]
			Suramin	[37]
			Melarsoprol	
			Eflornithine	[38]
			Nifurtimox	
hsa05133:Pertussis	P28482, P29460, P35228, P01375	0.0058445	Erythromycin	
hsa05164:Influenza A	P28482, P31749, P29460, P05362, P01375	0.0101454	**Oseltamivir**	[18]
hsa05146:Amoebiasis	P29460, P35228, P02751, P01375	0.0150504	Metronidazole	[28]
hsa05168:Herpes simplex infection	P04637, P29460, Q9NR96, P01375	0.0606792	**Acyclovir**	[18]
			Famciclovir	
**Annotation Cluster 4 (Enrichment Score: 2.23)**				
**Term**	**Associated proteins**	**P-value**	**FDA Approved**	**Reference**
hsa05212:Pancreatic cancer	P15692, P04637, P28482, P31749, P00533, P40763	1.45E-05	Capecitabine	
			Fluorouracil	

## Conclusion and discussion

Drug repurposing is a beneficial field of research and its importance has been increasing in the past years. This field has several advantages. For example, it makes the clinical trial procedure shorter. It also helps in discovering previously unknown relationships between diseases. The urgent need to find effective drugs for COVID-19 has hardly pushed this area of research in the past months. Computational methods play an important role to find effective drugs among available drugs for COVID-19 treatment. One of the best ways to identify effective drugs in different diseases is to find disease pathways related to the pathology of diseases. Most of the drug repurposing methods are based on finding biological properties for drug targets. Therefore, the main idea of this paper is to find a set of disease pathways related to the pathology of COVID-19 that can help us find some appropriate drugs for COVID-19 treatment. For this purpose, we proposed a method that used disease-associated genes, biological properties that are affected by the virus, topological and statistical properties. In the first part of our method, we defined 4 informative topological features and 4 informative statistical features for each protein reported by Uniprot as a drug target. Our results for the first part suggested a set of proteins that have valuable topological and biological properties compared to other protein sets with respect to the number of Covid-Drugs and Clinical-Drugs approved by this candidate set. In the second part of this work, we studied genes associated with some underlying diseases to identify a subset of genes related to COVID-19 pathology. These underlying diseases were cardiovascular diseases, diabetes, hepatitis, lung diseases, kidney disease, and different cancer types. Our results for the second part presented 35 genes associated with at least four of five underlying mentioned diseases as genes related to COVID-19 pathology. The resulted genes from this part of our method are evaluated with respect to different measures. The first measure was based on drug targets. We found that from these 35 genes 9 genes are targeted by the Covid-Drug group and 24 genes are targeted by the Clinical-Drug group. The second measure was based on the related significant signaling pathways related to COVID-19. We explored that some pathways like the **HIF-1** signaling pathway or **PI3K-Akt** signaling pathway are affected by the SARS-CoV-2 virus. It is noticeable that, From 56 drugs recommended through our method, 26 drugs are reported in other studies as possible candidates for COVID-19 drug repurposing. 7 drugs **Cyclosporine, Ribavirin, Enzalutamide, Decitabine, Imatinib, Metformin, Oseltamivir**, and **Acyclovir** are reported in DrugBank that are under clinical trials for COVID-19 treatment. It can be concluded that other drugs reported through our method can be effective and suitable candidates. They could be good candidates for further research in clinical trials for COVID-19 treatment.
